# c-FLIP regulates autophagy by interacting with Beclin-1 and influencing its stability

**DOI:** 10.1038/s41419-021-03957-5

**Published:** 2021-07-08

**Authors:** Luana Tomaipitinca, Simonetta Petrungaro, Pasquale D’Acunzo, Angelo Facchiano, Amit Dubey, Salvatore Rizza, Federico Giulitti, Eugenio Gaudio, Antonio Filippini, Elio Ziparo, Francesco Cecconi, Claudia Giampietri

**Affiliations:** 1 Department of Anatomy, Histology, Forensic Medicine and Orthopedics, Sapienza University of Rome, Rome, Italy; 2 Cell Stress and Survival Unit, Danish Cancer Society Research Center, Copenhagen, 2100 Denmark; 3 Center for Dementia Research, Nathan S. Kline Institute for Psychiatric Research, Orangeburg, NY 10962 USA; 4 Department of Psychiatry, New York University School of Medicine, New York, NY 10016 USA; 5 Istituto di Scienze dell’Alimentazione-CNR, Avellino, Italy; 6 Computational Chemistry and Drug Discovery Division, Quanta Calculus Pvt Ltd, Kushinagar, 274203 India; 7 Department of Pharmacology, Saveetha Dental College and Hospital, Saveetha Institute of Medical and Technical Sciences, Chennai, Tamil Nadu India; 8 Redox Signaling and Oxidative Stress Group, Danish Cancer Society Research Center, Copenhagen, 2100 Denmark; 9 Department of Pediatric Hemato-Oncology and Cell and Gene therapy, IRCCS Bambino Gesù Children’s Hospital, Rome, 00143 Italy; 10 Department of Biology, University of Tor Vergata, Rome, 00133 Italy

**Keywords:** Biochemistry, Autophagy

## Abstract

c-FLIP (cellular FLICE-like inhibitory protein) protein is mostly known as an apoptosis modulator. However, increasing data underline that c-FLIP plays multiple roles in cellular homoeostasis, influencing differently the same pathways depending on its expression level and isoform predominance. Few and controversial data are available regarding c-FLIP function in autophagy. Here we show that autophagic flux is less effective in *c-FLIP−/−* than in WT MEFs (mouse embryonic fibroblasts). Indeed, we show that the absence of c-FLIP compromises the expression levels of pivotal factors in the generation of autophagosomes. In line with the role of c-FLIP as a scaffold protein, we found that c-FLIP_L_ interacts with Beclin-1 (*BECN1*: coiled-coil, moesin-like BCL2-interacting protein), which is required for autophagosome nucleation. By a combination of bioinformatics tools and biochemistry assays, we demonstrate that c-FLIP_L_ interaction with Beclin-1 is important to prevent Beclin-1 ubiquitination and degradation through the proteasomal pathway. Taken together, our data describe a novel molecular mechanism through which c-FLIP_L_ positively regulates autophagy, by enhancing Beclin-1 protein stability.

## Introduction

FLIP (FLICE-like inhibitory protein) was described for the first time as a viral factor utilized by pathogens to escape apoptotic signalling in host cells [1]. FLIP homologues were then identified in human cells and referred to as cellular FLIP (c-FLIP) [2]. Since their characterization as anti-apoptotic factors, it became clear that c-FLIP family members exert multifunctional roles in several cellular processes, as they are involved in embryonic development, cardiac hypertrophy, skeletal muscle homoeostasis, T-cell proliferation and activation of nuclear factor-κ-light-chain-enhancer of activated B cells and extracellular signal-regulated kinase pathways [3–7]. Apoptosis plays a key role in liver toxic injury and its enhancement via c-FLIP removal increases liver damage in vivo [8].

Thirteen distinct splice variants originate from the human *CFLAR* gene, but only three of them are translated into proteins: the long isoform c-FLIP_L_ (55 kDa) and two short isoforms, namely c-FLIP_R_ (25 kDa) and c-FLIP_S_ (27 kDa) [2]. In mice, only two c-FLIP isoforms are expressed: a long and a short isoform [9]. c-FLIP structure contains two death effector domains, which mediate c-FLIP recruitment at the death-inducing signalling complexes, thus competing with procaspase 8 and inhibiting apoptosis [2]. Although the short isoforms have been confirmed to only act as negative regulators of cell death, the role of c-FLIP_L_ in the balance between cell death and cell survival is more intricated and controversial. Numerous lines of evidence show that c-FLIP_L_ can also support apoptosis activation [10].

Differences between c-FLIP isoforms also concern their cellular localization. Although all three variants can be found in the cytosol, c-FLIP_L_ is the only one that can also localize at the endoplasmic reticulum (ER), at the mitochondria associated membranes (MAMs) and in the nucleus [11, 12].

It is emerging that c-FLIP exerts pro- or anti-apoptotic functions depending on its expression levels, and that c-FLIP_L_ contribution to cell death is strictly cell type-dependent and rely on relative levels of its isoforms. Indeed, the balance between all c-FLIP isoforms controls cell fate in a dose-dependent way [13, 14].

Macroautophagy (hereafter referred to as autophagy) is a highly conserved catabolic mechanism that ensures quality control of macromolecules and whole organelles. Autophagy contributes to cellular homoeostasis by regulating the turnover of cellular components and plays a pivotal role in the regulation of body metabolism [15, 16]. Autophagy is involved in several biological processes, such as embryonic development and cellular differentiation, but also in stress-inducing responses, which enhance autophagy activation. This mechanism is triggered by nutrients deprivation, oxidative stress and pathogen invasion, and it is impaired in several human pathologies, including neurological and metabolic disorders, infections, autoimmune diseases and tumorigenesis [17–23].

When autophagy is induced, cytoplasmic regions to be targeted for degradation are incorporated into a system of forming membranes, called phagophores or isolation membranes. The expansion of the phagophores leads to closed vesicular structures, namely autophagosomes, which are transported along the microtubules to fuse with lysosomes and form the autophagolysosomes, where hydrolytic enzymes catalyse cargo degradation [24–28].

c-FLIP isoforms were also associated with autophagy. First, it has been reported that c-FLIP can prevent autophagy-related gene 3 (Atg3) from binding microtubule-associated protein light-chain 3 (LC3). As Atg3-mediated LC3 processing and lipidation is fundamental for autophagosome formation, it was proposed that c-FLIP suppresses autophagy in basal conditions [29]. On the other hand, the antagonist of c-FLIP caspase 8 was found to play a role in autophagy repression in T cells. In detail, Fas-associated protein with death domain–caspase 8 complex can localize at the expanding autophagosome, an event that leads to caspase 8 activity induction, resulting in autophagic cell death inhibition. In the same work, the authors hypothesize that differential expression of c-FLIP isoforms can modulate the activation of caspase 8 during autophagy [30]. Remarkably, other reports show a positive correlation between the overexpression of the short c-FLIP isoform (c-FLIP_S_) and pro-autophagic vesicle formation during anti-tumour treatments [31].

We previously demonstrated that c-FLIP absence alters ER stress-dependent apoptosis in mouse embryonic fibroblasts (MEFs), promoting cell survival [32]. Considering that autophagy is one of the most important pro-survival processes activated by cells to cope with stress conditions [33], we decided to investigate the effects of c-FLIP on autophagy activation. In the present study, we show that an interplay does exist between c-FLIP_L_ and Beclin-1 (*BECN1*: coiled-coil, moesin-like BCL2-interacting protein). Beclin-1, along with Vps34 (vacuolar protein sorting) and Vps15, constitute the phosphatidylinositol-3-kinase (PI3K-III) core complex, which mediates the formation of phosphatidylinositol-3-phosphate (PtdIns3P), a signal molecule required for membrane specialization into phagophores [26]. Beclin-1 is essential for the association of the core complex to several transient interactors, important to define the complex localization and its biological activity [34–37]. By connecting the factors modulating the kinase activity of Vps34 to their target kinase, Beclin-1 stands at the centre of PI3K-III complexes’ regulation and it is widely monitored. Beclin-1 can undergo several post-translational modifications, such as phosphorylation, ubiquitination, ISGylation and acetylation, consequently affecting the autophagic process [37]. Reduced expression of Beclin-1 was found in hepatocarcinoma, as well as in intrahepatic and extrahepatic cholangiocarcinoma, indicating Beclin-1 as a possible prognosis marker [38, 39]. We here unravel a new molecular mechanism involved in Beclin-1 protein level control, demonstrating that c-FLIP_L_ prevents Beclin-1 proteasome-mediated degradation by decreasing Beclin-1 ubiquitination state and acts as a positive regulator of autophagic flux.

## Results

### Autophagic flux is compromised in *c-FLIP−/−* cells

**Fig. 1:**
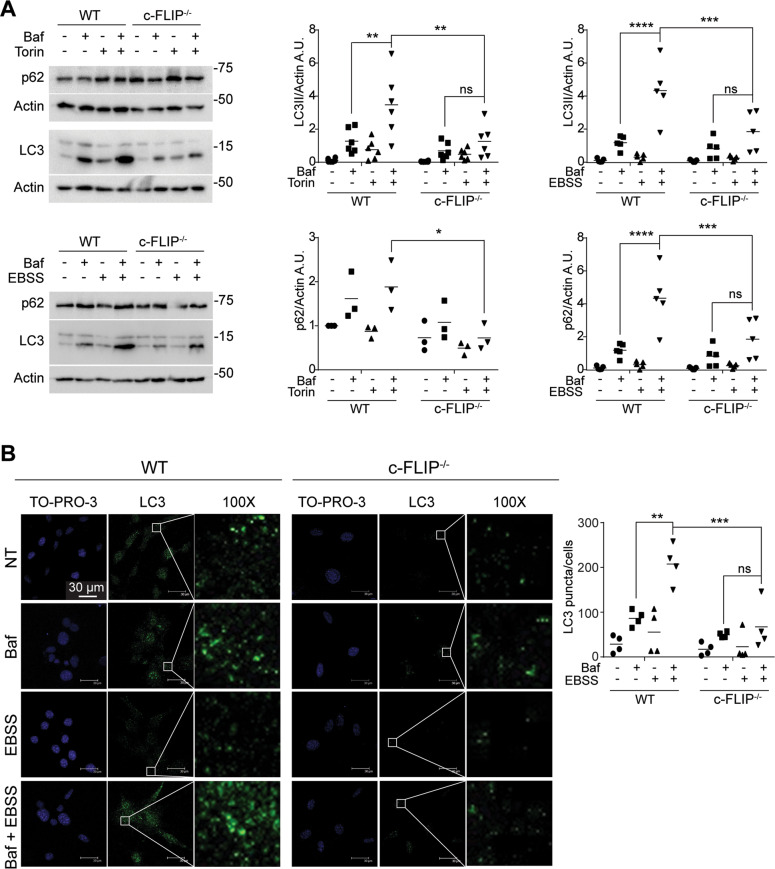
Autophagic flux is compromised in *c-FLIP−/−* cells. **A** WT and *c-FLIP−/−* MEFs were treated with torin 1 (Tor) (250 nM) and bafilomycin A1 (Baf) (100 nM) or cultured in a starvation medium (EBSS) and treated with bafilomycin A1 (Baf) (100 nM) for 3 h. Autophagic flux was assessed by measuring LC3 II and p62 protein levels by western blotting. β-Actin was used as a loading control. Data shown are representative of at least three individual experiments. *p < 0.05, **p < 0.01, ***p < 0.001, ****p < 0.0001 determined by two-way ANOVA. **B** WT and *c-FLIP−/−* MEFs were cultured in EBSS and treated with bafilomycin A1 for 3 h. Cells were fixed and stained with anti-LC3 antibody (green) and then analysed by confocal microscopy. The images and the graph shown are representative of at least three individual experiments. **p < 0.01, ***p < 0.001 determined by two-way ANOVA. Scale bar, 30 μm.

We previously demonstrated that the absence of c-FLIP compromises MEFs response to ER stress-dependent cell death induced by tunicamycin. [32]. As ER stress response is strictly connected to autophagy activation, we wondered whether autophagic flux was altered in *c-FLIP−/−* MEFs [40]. To study c-FLIP-dependent autophagic flux, we decided to analyse two well-known autophagy markers, LC3 and p62, in wild-type (WT) MEFs compared to *c-FLIP−/−* MEFs. As autophagy is a highly rapid answer to stress, and LC3 II and p62 turnover may be very fast, as already described, we used bafilomycin A1, an inhibitor of autophagosomes and lysosomes fusion, to block LC3 II and p62 degradation into autophagosomes [41]. Tunicamycin induced a stronger autophagy activation in WT compared to *c-FLIP−/−* MEFs, as detected by LC3 II and p62 accumulation in bafilomycin A1-treated cells (Supplementary Fig. S1). To specifically investigate c-FLIP involvement in autophagy, we induced the mechanism in our cells using two more stimuli, widely known to trigger this process: starvation, obtained by replacing full medium with Earle’s balanced salt solution (EBBS), or torin 1 treatment, a specific mammalian target of rapamycin (mTOR) inhibitor [41]. In both starvation and torin treatment (Fig. 1A), in the presence of bafilomycin A1, we observed the accumulation of LC3 II and p62 only in WT MEFs, whereas no significant markers accumulation was observed in *c-FLIP−/−* MEFs.

To further corroborate these results, autophagic flux in starvation conditions was measured by immunofluorescence, by analysing the appearance of LC3-positive puncta, in bafilomycin A1-treated cells. Coherently with western blotting outcomes, quantification of LC3 puncta shows stronger accumulation in WT cells than in *c-FLIP−/−* cells (Fig. 1B).

Finally, we checked Atg5 expression levels in starvation conditions at different time points. Atg5 belongs to the Atg5–Atg12–Atg16 complex, required for LC3 lipidation and maturation [42]. As shown in Supplementary Fig. S2, Atg5 protein expression increases following nutrient deprivation in WT MEFs, whereas no significant differences can be observed in *c-FLIP−/−* cells.

This evidence leads us to conclude that c-FLIP is required to carry on autophagic flux.

### c-FLIP absence does not influence autophagy induction

Based on the previous results, to understand whether the absence of c-FLIP affected autophagy by preventing the induction step of the process, we studied mTOR and Unc-51-like autophagy activating kinase (ULK1) activation state under starvation conditions at increasing time points.

**Fig. 2:**
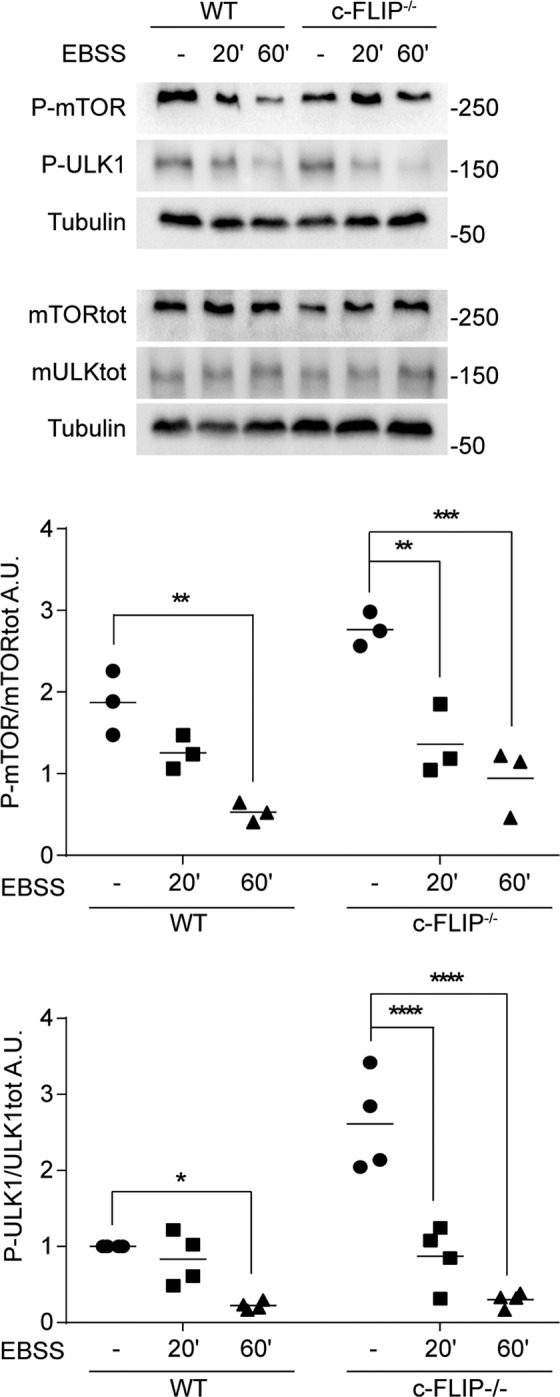
WT and *c-FLIP−/−* MEFs were cultured in EBSS for increasing times (20 and 60 min). Total mTOR protein and its phosphorylation levels on Ser2448, and total ULK1 and its phosphorylation levels on Ser757 were evaluated by western blotting. Graphs show the ratio of P-mTOR on mTOR total protein and of P-ULK1 on ULK1 total protein. α-Tubulin was used as a loading control. Data shown are representative of at least three individual experiments. *p < 0.05, **p < 0.01, ***p < 0.001, ****p < 0.0001 determined by two-way ANOVA.

When nutrient levels available are sufficient, mTOR is activated by numerous post-translational modifications, including a positive feedback mechanism based on phosphorylation by its direct target S6 kinase 1 at Ser2448 [43]. These phosphorylation levels rapidly attenuate after nutrient deprivation, leading to mTOR deactivation and removing mTOR-mediated autophagy blockage. As evident in Fig. 2, starvation reduced mTOR Ser2448 phosphorylation in both WT and *c-FLIP−/−* MEFs, indicating autophagy activation in both cell types.

This result was confirmed by studying ULK1, which is a direct target of mTOR. Indeed, mTOR phosphorylates ULK1 on Ser757 to inhibit autophagy [44]. After starvation, the phosphorylation on ULK1 Ser757 decreases in both WT and *c-FLIP−/−* MEFs (Fig. 2), demonstrating that the absence of c-FLIP does not affect the induction phase of the process.

### c-FLIP absence impacts the expression levels of key proteins in autophagosomes nucleation

As we did not find any significant differences in the induction of autophagy depending on c-FLIP absence, we focused on the nucleation step of the process, in particular on the members of the PI3K-III complex, which mediates PtdIns3P synthesis.

We thus evaluated by western blotting the levels of Beclin-1, Vps34 and the phosphorylation status of Atg14 after starvation in a 3 h timeframe. Beclin-1 acts as an adaptor protein, which associates to the phosphatidylinositol-3-kinase Vps34 regulating its cognate partners binding. This interaction is crucial to constitute the nucleation core complex and for the kinase to generate PtdIns3P [37]. Atg14 is another partner of the complex, ensuring its correct localization at the ER. It is phosphorylated by ULK1 at Ser29 and this phosphorylation rate increases upon autophagy-inducing stimuli [45].

**Fig. 3:**
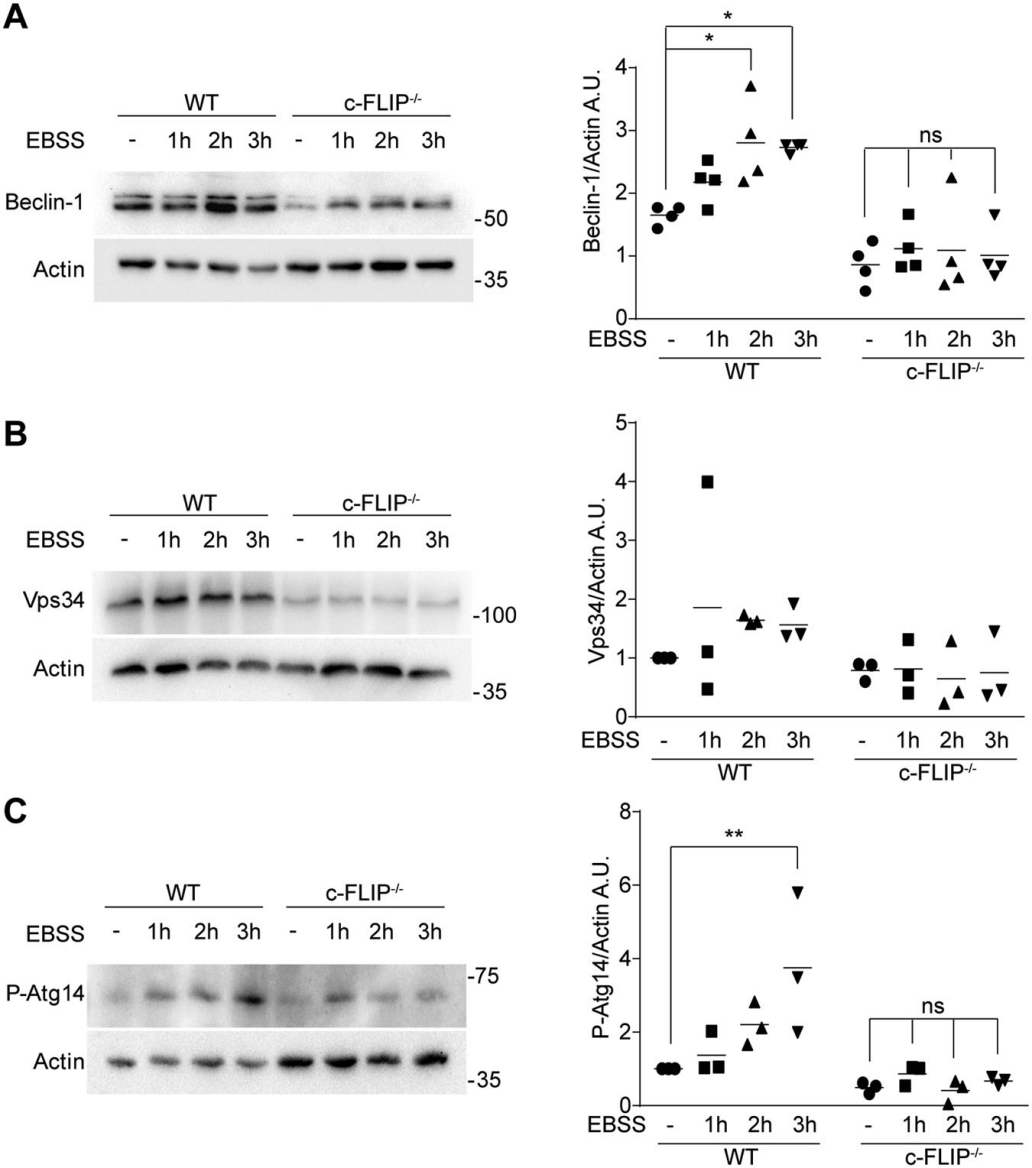
WT and *c-FLIP−/−* MEFs were cultured in EBSS for increasing times (1, 2 and 3 h) and the expression levels of Beclin-1, Vps34 and P-Atg14 were analyzed. **A** Beclin-1, **B** Vps34 and **C** P-Atg14 on Ser29 were studied by western blotting. β-Actin was used as a loading control. Data shown are representative of at least three individual experiments. *p < 0.05, **p < 0.01 determined by two-way ANOVA.

As displayed in Fig. 3A–C, nutrient deprivation results in a time-dependent increase in the expression levels of these proteins in WT MEFs. Interestingly, c-FLIP_L_ levels are not influenced by starvation (Supplementary Fig. S3A). On the contrary, in *c-FLIP−/−* MEFs, starvation triggers a milder effect on these factors. Remarkably, the expression levels of Beclin-1 result to be lower in *c-FLIP−/−* MEFs, compared to WT cells also in untreated conditions, with a significant mean difference of 52% (Supplementary Fig. S3B).

These data indicate that c-FLIP presence is pivotal for autophagic membrane nucleation process in response to starvation.

### c-FLIP_L_ interacts with Beclin-1 and affects Beclin-1 degradation

As c-FLIP is a scaffold protein without known catalytic activities, it participates to cellular processes by binding other proteins and either assembling or destabilizing complexes. Therefore, we wondered whether c-FLIP could physically interact with components of the nucleation core.

It is known that Beclin-1 is a direct target of caspase 8, and that this enzyme inactivates autophagy by cleaving Beclin-1 [46]. As caspase 8 shows high structural homology with c-FLIP and the roles of these two proteins in cellular homoeostasis are strictly connected [10], we hypothesized that Beclin-1 could be a possible interactor of c-FLIP.

**Fig. 4:**
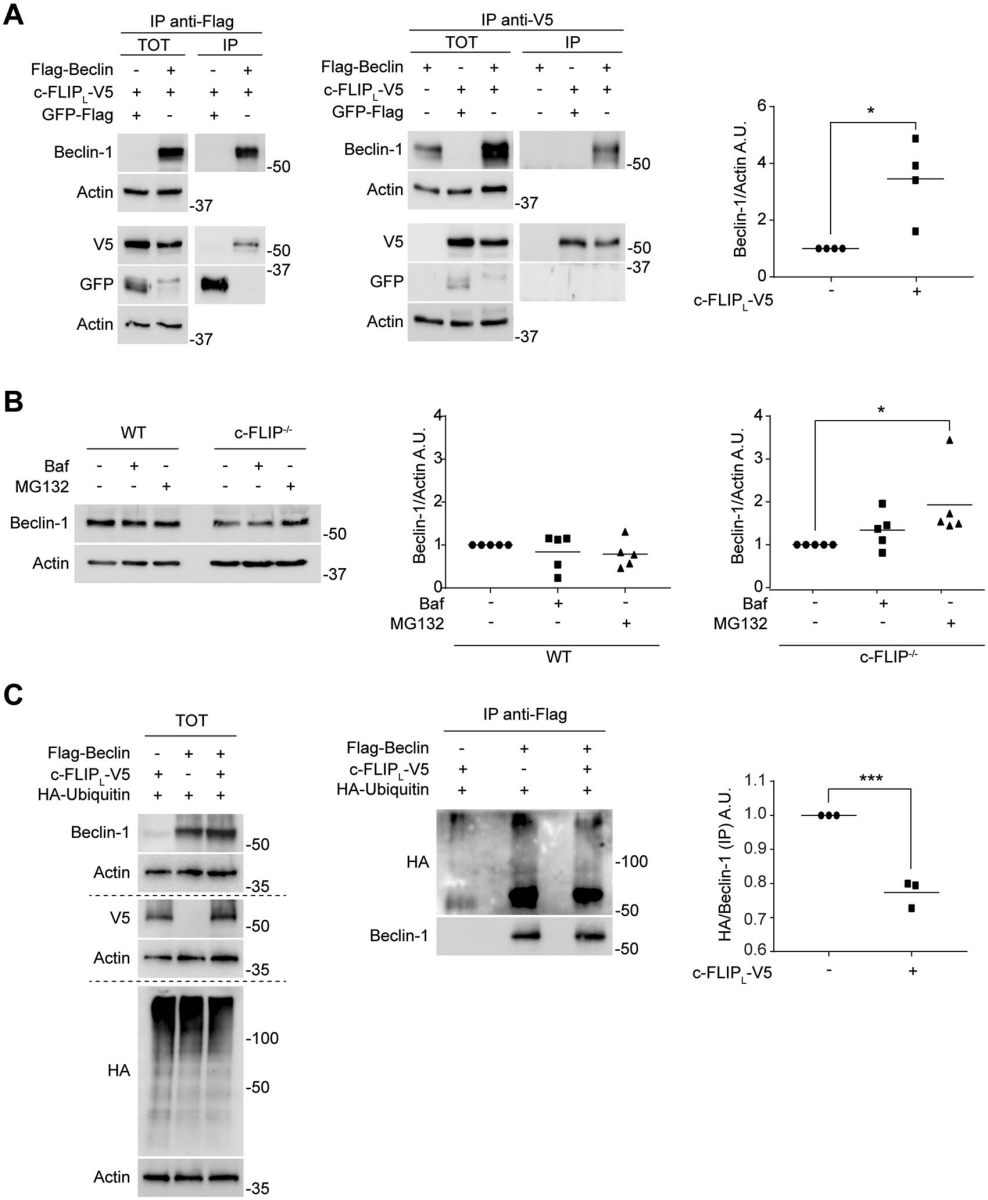
c-FLIP_L_ interacts with Beclin-1 and affects Beclin-1 degradation. **A** Hek293 cells were transfected to overexpress c-FLIP (c-FLIP_L_-V5) and Beclin-1 (Flag-Beclin). α-Flag-beads were used to immunoprecipitate Beclin-1 and an α-V5 antibody was used to immunoprecipitate c-FLIP. GFP-Flag was transfected to verify the specificity of the interaction. The result was detected by western blotting. β-Actin was used as a loading control. The graph quantifies Beclin-1 expression levels in correlation with c-FLIP overexpression. Data shown are representative of at least three individual experiments. *p < 0.05 determined with two-tailed Student’s *t*-test. **B** WT and *c-FLIP−/−* MEFs were treated with either bafilomycin (Baf) (100 nM) or MG132 (10 μM) for 4 h and Beclin-1 protein expression was then analysed by western blotting. Data shown are representative of at least three individual experiments. β-Actin was used as a loading control. *p < 0.05 determined by one-way ANOVA. **C** Hek293 cells were transfected to overexpress c-FLIP (c-FLIP_L_-V5), Beclin-1 (Flag-Beclin) and ubiquitin (HA-Ubiquitin). α-Flag-beads were used to immunoprecipitate Beclin-1. Protein signals were evaluated via western blotting and β-Actin was used as a loading control. Ubiquitination of Beclin-1 (HA/Beclin-1) was quantified in the graph. Data shown are representative of three individual experiments. ***p < 0.001 determined with two-tailed Student’s *t*-test.

To investigate this issue, HEK 293 cells were transiently transfected with two tagged constructs expressing Flag-Beclin and c-FLIP_L_-V5. We performed two co-immunoprecipitation assays on the cellular extracts and found that these two proteins interact (Fig. 4A). We also checked for an interaction between Beclin-1 and FLIP_S_ by immunoprecipitation of c-FLIP isoforms. We detected a very low signal (if any) for Beclin-1, suggesting the absence of any biologically relevant binding between Beclin-1 and the short isoform of c-FLIP (Supplementary Fig. S4).

Interestingly, western blot analysis of HEK 293 total extracts revealed higher levels of Beclin-1 in cells in which Flag-Beclin was co-transfected with c-FLIP_L_ compared to cells in which Flag-Beclin was the only protein overexpressed (significant fold increase mean of 3.45) (Fig. 4A). This observation, together with data shown in Fig. 3 and Supplementary Fig. S3, where it is evident how Beclin-1 and the other nucleation proteins expression levels are lower in *c-FLIP−/−* compared to WT MEFs even in endogenous and basal conditions, suggested us that c-FLIP could be involved in the post-translational regulation of Beclin-1.

To verify our hypothesis, we treated both WT and *c-FLIP−/−* MEFs with MG132, which binds the active site of the 26S proteasome complex, thus blocking protein degradation [47] or bafilomycin A1 to prevent autophagosomes turnover. Indeed, Beclin-1 significantly accumulates with MG132 in *c-FLIP−/−* MEFs but not when using bafilomycin, whereas no differences can be observed in WT cells between the treated and the untreated samples (Fig. 4B). Consequently, we wondered if Beclin-1 ubiquitination state was influenced by c-FLIP_L_. To address this issue, we transfected HEK 293 cells with a Flag-Beclin-expressing plasmid with or without a second plasmid expressing c-FLIP_L_-V5. We next performed an immunoprecipitation assay to isolate Beclin-1 protein and found that its ubiquitination levels significantly decrease in dependence of c-FLIP_L_ overexpression (Fig. 4C), leading us to conclude that c-FLIP_L_ is important to stabilize Beclin-1 protein expression by reducing Beclin-1 proteasomal degradation.

### Bioinformatics simulation and biochemical data correlate with a competition dynamic between c-FLIP_L_ and NEDD4

**Fig. 5:**
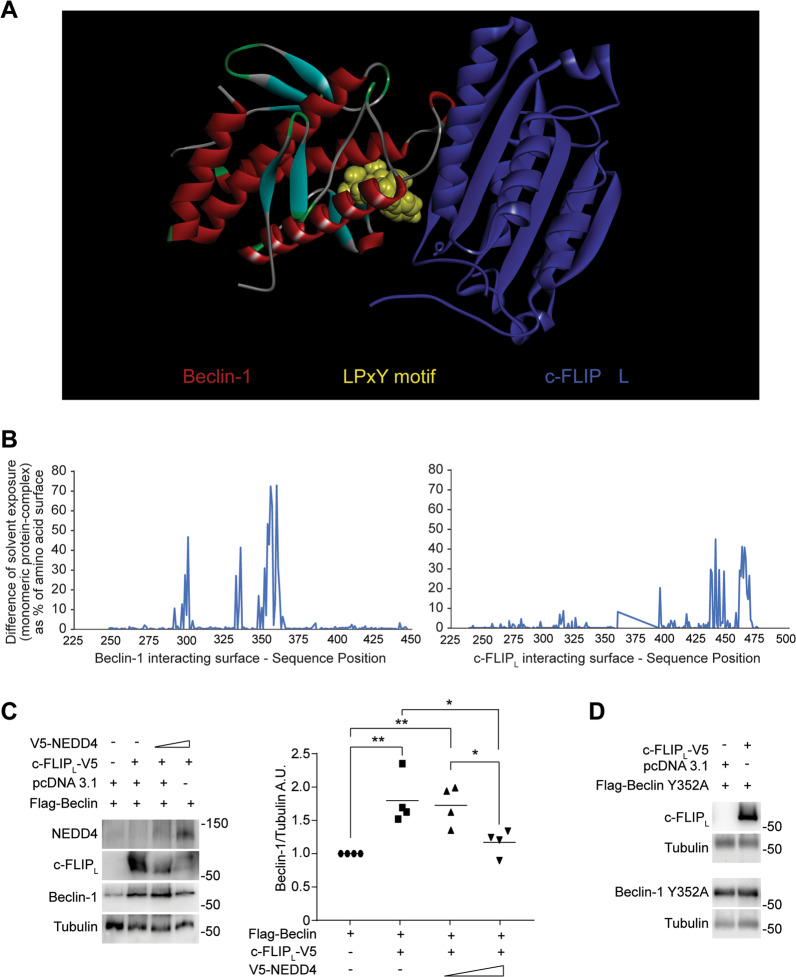
Bioinformatics simulation and biochemical data correlate with a competition dynamic between *c-FLIP*_*L*_ and NEDD4. **A** The interaction between Beclin-1 and c-FLIP was simulated. Beclin-1 (on the left) is shown as a backbone ribbon with standard colours (red = helices, cyan = β-strand, green = turn). The 349–352 region, which is reported in literature as being involved in the interaction with NEDD4, is highlighted in yellow. The region is near the surface of interaction with the c-FLIP molecule (on the right), shown with backbone ribbon in blue. **B** The graph evidences the sequence regions of Beclin-1 and of c-FLIP involved into the interaction between the two molecules as it reports the difference of solvent exposure of amino acids from the monomeric to the complex structure. Values > 0 indicate amino acids that are solvent exposed in the monomeric structure and are not accessible, or less accessible, to the solvent in the complex, i.e., at the interface with the interacting molecule. **C** Hek293 cells were transfected to overexpress c-FLIP (c-FLIP_L_-V5), Beclin-1 (Flag-Beclin) and NEDD4 (V5-NEDD4). Beclin-1 protein expression was then detected by western blotting. α-Tubulin was used as a loading control. Data shown are representative of at least three individual experiments. *p < 0.05, **p < 0.01 determined by one-way ANOVA. **D** Hek293 cells were transfected to overexpress c-FLIP (c-FLIP_L_-V5) and Beclin-1 mutant (Flag-Beclin Y352A). Beclin Y352A protein expression was studied by western blot analysis. α-Tubulin was used as a loading control. Data shown are representative of three individual experiments.

As mentioned above, Beclin-1 is a pivotal member of the PI3K-III complex and its expression levels are tightly regulated via post-translational modifications, which influence its activity on autophagy [37]. We thus used bioinformatic tools to simulate Beclin-1/c-FLIP_L_ interaction and found that the amino acids 349–352 of Beclin-1 are located at the interface region in the theoretical c-FLIP_L_/Beclin-1 complex, as shown in Fig. 5A. The 349–352 region constitutes Beclin-1 PY motif (LPxY), which is a target of NEDD4 (neural precursor cell-expressed developmentally downregulated 4), a ubiquitin ligase that was shown to influence autophagy at multiple levels by interacting with several autophagic factors, including Vps34, p62 and Beclin-1 [48–51]. In particular, NEDD4 binds Beclin-1 via its PY motif, thus catalysing Beclin-1 polyubiquitination, an event that causes Beclin-1 degradation via the proteasome [52]. The position of the PY motif at the interface is also confirmed by the comparison of surface analysis on the monomeric structure and the complex (Fig. 5B). By studying, the solvent exposure of amino acids along the sequence revealed that the region 349–352 (and the following) in the complexed structure is different than in the monomeric structure. In more detail, this region results to be less accessible to the solvent in the complex than in the monomeric structure. This suggests that the predicted interaction of c-FLIP_L_ with Beclin-1 may mask the 349–352 segment, thus preventing Beclin-1 interaction with NEDD4. Moreover, on the other side of the complex, c-FLIP_L_ interacts with Beclin-1 via c-FLIP_L_ C-terminal portion, in particular via the segment formed by amino acids 463–479. To verify the hypothesis suggested by the bioinformatic analysis, we addressed c-FLIP_L_-NEDD4 competition dynamic by transiently transfecting HEK 293 cells with c-FLIP_L_-V5- and V5-NEDD4-expressing plasmids, and evaluating their effect on Beclin-1 protein stability. As shown in Fig. 5C, c-FLIP_L_ transfection enhances Beclin-1 protein levels as we previously showed. Combined c-FLIP_L_-NEDD4 transfection affects Beclin-1 levels, depending on the ratio of c-FLIP_L_/NEDD4 levels. As this ratio decreases, so does Beclin-1 expression level, therefore supporting the hypothesis that c-FLIP_L_ prevents NEDD4/Beclin-1 binding, thus reducing Beclin-1 proteasomal degradation. To further investigate this issue, we mutated Tyr352 of Beclin-1 into alanin (Y352A) according to previous evidence showing this amino acid substitution as capable to reduce Beclin-1 interaction with NEDD4 and consequently its ubiquitination [52]. Our experiments show that Beclin-1 Y352A mutant was, indeed, unaffected by c-FLIP_L_ transfection (Fig. 5D), indicating that Tyr352 is required for c-FLIP_L_-dependent Beclin-1 stabilization and corroborating a competition dynamic between c-FLIP_L_ and NEDD4.

## Discussion

In recent years, it became clear that the contribution of c-FLIP to cellular homoeostasis is complex, as c-FLIP controls different processes [13, 53, 54]. The involvement of c-FLIP in autophagy is a recent finding, which requires a deeper elucidation. In this work, we provide evidence for a direct influence of c-FLIP long isoform on autophagy.

Indeed, we here demonstrate that MEFs, which do not express c-FLIP, display a compromised autophagic flux in response to autophagy-inducing stimuli compared to WT cells (Fig. 1). Interestingly, when studying the autophagy induction stage, we found that both cell lines can trigger the process as mTOR kinase, the main endogenous inhibitor of autophagy, is inactivated upon starvation in both WT and *c-FLIP−/−* MEFs [43]. Analysis of the activation state of ULK1, a direct target of mTOR-mediated inhibitory phosphorylation, following a pro-autophagic stimulus confirmed that autophagy initiation happens regardless of c-FLIP protein presence (Fig. 2) [44].

Our group characterized c-FLIP as a modulator of ER morphology and ER–mitochondria crosstalk, showing that c-FLIP_L_ localizes at ER and MAMs, and that *c-FLIP−/−* MEFs display an enlarged ER structure [12]. Remarkably, both ER and MAMs have been associated to omegasomes, the membranous structures from which autophagic vesicles originate [24]. Upon autophagy induction, the molecular machinery responsible for autophagosome nucleation migrates to omegasomes, where it mediates the production of PtdIns3P and induces membranes specialization into phagophores [26]. Thus, we investigated Beclin-1, Vps34 and P-Atg14 expression upon starvation in dependence of c-FLIP. Selective binding of Atg14 to the core nucleation complex (Beclin-1-Vps34-Vps15) mediates its presence at the ER in the autophagosomes nucleation step [37]. We found that the levels of all the three markers increase after autophagy induction in WT MEFs, as expected, but not in *c-FLIP−/−* cells. We also found that all these proteins are downregulated in *c-FLIP−/−* cells compared to WT in basal conditions as well (Fig. 3 and Supplementary Fig. S3).

c-FLIP is a scaffold protein devoid of any catalytic activity [10], which suggests that it may interact with the proteins involved in the autophagosome nucleation phase. All factors that participate to this step of the autophagic process are highly regulated and strongly dependent on each other. In fact, the depletion of even only one of them compromises the entire complexes, as feedback mechanisms are called in action [26].

We here demonstrate that the absence of c-FLIP results in an alteration of the expression levels of some nucleation factors, i.e., Beclin-1 in particular (Fig. 3 and Supplementary Fig. S3). Therefore, we next explored a possible interaction between c-FLIP_L_ and Beclin-1. The co-immunoprecipitation assays we performed shows that the two proteins interact in basal conditions. Furthermore, we noticed that Beclin-1 expression levels are affected by c-FLIP_L_, both in endogenous and in overexpressing conditions in two different cellular models, suggesting that c-FLIP_L_ could influence Beclin-1 protein at a post-translational level. To investigate this issue, we treated WT and *c-FLIP−/−* MEFs with MG132, a drug that blocks proteasome activity. In this way, we increased Beclin-1 levels in *c-FLIP−/−* cells, whereas proteasomal blockage did not affect the protein level in WT cells, suggesting that the lack of c-FLIP accelerates Beclin-1 turnover via proteasome. Furthermore, we show that c-FLIP_L_ overexpression induces a reduction in Beclin-1 ubiquitination state, corroborating the theory of a highest degradation rate of Beclin-1 upon c-FLIP depletion and, therefore, that c-FLIP expression favours Beclin-1 protein stability (Fig. 4).

Finally, information obtained from our bioinformatic simulation suggested that the interaction between the C-terminal portion of c-FLIP_L_ and Beclin-1 involves Beclin-1 amino acids 349–352, which constitute the PY motif, recognized by the E3 ubiquitin ligase NEDD4 to label Beclin-1 for proteasomal degradation [52]. Interestingly, the C-terminal region of c-FLIP_L_ (amino acids from 463 to 479) is present only in the c-FLIP long isoform. These data well correlate with our biochemical analysis, which reveals a strong interaction between Beclin-1 and the long isoform of c-FLIP, but not with the short one (Fig. 4 and Supplementary Fig. S4). Therefore, c-FLIP_L_ interaction with Beclin-1 may mask pivotal residues to the recognition of Beclin-1 by NEDD4 (Fig. 5A, B). Indeed, biochemical analyses revealed that NEDD4 overexpression counteracts c-FLIP_L_ effect on Beclin-1 stability, causing a decrease in Beclin-1 protein levels in a dose-dependent way (Fig. 5C). Moreover, Beclin-1 mutation of Tyr352 to alanine, which reportedly diminishes Beclin-1 ubiquitination and degradation via NEDD4, also prevents c-FLIP_L_ effects on Beclin-1 stability, as we did not observe a difference of the Y352A mutant in dependence of c-FLIP_L_ overexpression (Fig. 5D). These results corroborated the concept of a competition dynamic between c-FLIP_L_ and NEDD4, which directly impacts Beclin-1 protein levels by regulating its turnover by the proteasomal pathway.

Our findings contribute to deepen the understanding of the complex crosstalk between autophagy and apoptosis. c-FLIP and Beclin-1 are key factors in the two pathways and they are both intriguing targets in anti-tumour therapies. As c-FLIP is traditionally considered as an anti-apoptotic protein, the goal is often to reduce its expression to induce apoptosis in cancer cells [53]. On the contrary, Beclin-1 is a haploinsufficient tumour suppressor, known to be downregulated in many cancer types [55]. As Beclin-1 poor expression favours neoplastic transformations and tumour progression, the correlation we provide between these two proteins may pave the way to novel therapeutic paths. As it was widely demonstrated, balancing autophagy activation in cancer cells can influence their responses to therapies [56]. c-FLIP_L_-Beclin-1–autophagy axis investigation may therefore open novel interesting scenarios to be exploited in cancer therapy.

## Materials and methods

### Cell cultures

WT and *c-FLIP−/−* MEFs were generous gifts of Tak W. Mak (Amgen Institute, Toronto, Canada). MEFs were cultured in Dulbecco’s modified Eagle’s medium (DMEM) (Sigma-Aldrich, Milano, Italy), enriched with 10% fetal bovine serum (Gibco™, Life Technologies Corporation, Grand Island, NY, USA), 2 mmol/l glutamine, 1 mmol/l sodium pyruvate and non-essential amino acids, in the presence of 100 U/ml penicillin and 100 μg/ml streptomycin, all purchased from Sigma-Aldrich.

HEK 293 cells were cultured in DMEM enriched with 10% fetal bovine serum in the presence of 100 U/ml penicillin and 100 μg/ml streptomycin.

Cells were maintained at 37 °C in a humidified 5% CO_2_ atmosphere. All cell lines were tested for mycoplasma contamination prior to use.

### Cell treatments

Cells were plated in 35 mm dishes for immunoblotting and immunofluorescence assays, and they were grown overnight. The day after plating, cells were treated for the indicated time. Torin 1 was purchased from Tocris Bioscience (Bristol, UK). Bafilomycin A1, tunicamycin, EBSS, 3-methyladenine and MG132 were purchased from Sigma-Aldrich.

### Cloning

pcDNA3.1-Flag-BECN1.Y352A plasmid was generated using the Quick Change site-directed mutagenesis kit (Stratagene) on pcDNA3.1-Flag-BECN1 plasmid using the following oligonucleotides: BECN1 Y352A_S, 5′-TGCCGTTAGCCTGTTCTGGGGGGTTGCG-3′; BECN1 Y352A_AS, 5′-CCCAGAACAGGCTAACGGCAGCTCCTTAGATTTGTC-3′. Oligonucleotides were purchased by TAG Copenhagen (Denmark). Plasmid integrity was assessed by agarose gel electrophoresis and sequence confirmed by sequencing (Eurofin Genomics, Germany).

### Plasmids and transfection

We used the following plasmids: Flag-Beclin, Flag-BeclinY352A, c-FLIP_L_-V5 (Thermo Fisher Scientific, Life Technologies Corporation), c-FLIP_S_-myc (Thermo Fisher Scientific, Life Technologies Corporation), GFP-Flag, HA-Ubiquitin, V5-NEDD4 and pcDNA 3.1. V5-NEDD4 was a generous gift from Professor Daniela Rotin (University of Toronto, Canada). The day before transfection, cells were seeded to get the desired number of 80% confluence plates. Twenty-four hours later, cells were transfected using Lipofectamine2000® (Invitrogen, San Giuliano Milanese, Italy) according to the manufacturer’s guide.

### Cell lysis and protein quantification

Cells were washed two times with pre-chilled phosphate-buffered saline (PBS) purchased from Sigma-Aldrich and were lysed.

In the case of MEFs, a commercial lysis Buffer 10× (Cell Signaling Technology, Danvers, MA, USA) complemented with 2% sodium dodecyl sulfate (SDS) and proteases’ inhibitors (Sigma-Aldrich) was used. Cells were also sonicated (Branson Ultrasonic, Carouge, Switzerland) for 10 s at 50% amplitude.

In the case of HEK 293 cells, two different receipts were used as follows: (i) for the Beclin-1-c-FLIP co-immunoprecipitation: 50 mM Tris-HCl pH 7.5, 150 mM NaCl, 1 mM dithiothreitol, 0.5% Triton, 1 mM EDTA pH 8, proteases and phosphatases inhibitors, all purchased from Sigma-Aldrich; (ii) for the ubiquitination and the competition assay: 50 mM Tris-HCl pH 7.5, 1% Triton, 0.25% Na-Deoxycholate, 0.1% SDS, 150 mM NaCl, 1 mM EDTA pH 8, 5 mM MgCl_2_, proteases and phosphatases inhibitors, all purchased from Sigma-Aldrich. Cells were then incubated for 30 min on ice.

Lysates from both cell lines were then centrifuged at 4 °C for 10 min at 13,000 × *g* to remove cell debris.

Protein concentration was determined by micro BCA assay (Pierce, Rockford, IL, USA) and samples were boiled at 95 °C for 10 min following Laemmli Buffer addition (0.04% Bromophenol blue, 40% Glycerol, 2% SDS, 20% β-mercaptoethanol, 250 mM Tris-HCl pH.6.8, all purchased from Sigma-Aldrich.

### Co-immunoprecipitation

Equal amounts of proteins (300 μg) were used to perform the experiments.

In the case of Flag-Beclin, proteins were incubated with pre-washed agarose beads conjugated with anti-Flag antibody (Sigma-Aldrich) for 1 h at 4 °C with rotation. In the case of c-FLIP_L_-V5, proteins were incubated with 0.25 μg of monoclonal anti-V5 antibody (Cell Signaling Technology) overnight at 4 °C with rotation. The next day, pre-washed magnetic beads (Dynabeads® Protein G, Novex®, Life Technologies Corporation) were added to the mix and incubated for 1 h at 4 °C with rotation. In the case of c-FLIP, proteins were incubated with 0.25 μg of monoclonal anti-FLIP antibody (Cell Signaling Technology) overnight at 4 °C with rotation. The next day, pre-washed agarose beads (GE Healthcare, Chicago, IL, USA) were added to the mix and incubated for 1 h at 4 °C with rotation.

Samples were washed four times by centrifugation (5 min, 2500 × *g*) with washing buffer (lysis buffer receipt without protease and phosphatase inhibitors) to collect the beads. The proteins bound to the beads were eluted in 26 μl of 2× Laemmli Buffer and boiled.

### Ubiquitination assay

Equal amounts of proteins (300 μg) were used to perform the experiments.

Proteins were incubated with pre-washed agarose beads conjugated with anti-Flag antibody for 1 h at 4 °C with rotation. After the lysis and prior incubation with anti-Flag antibody, 1% SDS was added to the lysates, which were incubated at 90 °C for 5 min to dissociate protein–protein interactions as previously described [57]. Immunoprecipitation buffer was added to the lysates to tenfold dilute the samples. The immunoprecipitation was then carried on as described above.

### Immunoblotting

Proteins were separated by SDS–polyacrylamide gel electrophoresis and transferred on either polyvinylidene fluoride membranes or nitrocellulose membranes (Amersham Bioscience, Piscataway, NJ, USA). Membranes were probed using the following antibodies: anti-p62 (Abcam, Cambridge, UK); anti-Atg5, anti-P-Atg14 (Ser29), anti-Beclin-1, anti-FLIP, anti-LC3, anti-NEDD4, anti-mTOR, anti-P-mTOR (Ser2448), anti-ULK1, anti-P-ULK1 (Ser757), anti-V5, anti-Vps34 (Cell Signaling Technology); anti-BECN1, anti-GFP, anti-HA (Santa Cruz Biotechnology, Heidelberg, Germany); and anti-β-Actin, anti-Tubulin (Sigma-Aldrich). Secondary antibodies were horseradish peroxidase-conjugated anti-mouse or anti-rabbit (Bio-rad, Hercules, CA, USA). Membranes were washed with Tris-buffered saline (Medicago, Danmarks-Berga, Uppsala, Sweden) with 0.1% Tween-20 (Sigma-Aldrich) and developed through the chemiluminescence system (Amersham Bioscience) on the ChemiDock image analyser (Bio-Rad), which was also used for densitometric quantifications.

### Immunofluorescence

Cells were washed in PBS and fixed with 3.7% paraformaldehyde (Electron Microscopy Sciences, Industry Road Hatfield, PA, USA), followed by permeabilization with 0.4% Triton X-100 (Sigma-Aldrich) in PBS for 5 min. The samples were then blocked with 5% goat serum (Sigma-Aldrich) plus 0.3% Triton X-100 for 1.5 h and then incubated overnight with the primary antibody rabbit anti-LC3 (Cell Signaling Technology) according to the supplier’s indications. Following three washes of 5 min each with PBS, secondary goat anti-rabbit IgG-FITC antibody (Sigma-Aldrich) was added to the samples for 1 h. TO-PRO-3 Iodide (642/661) (Thermo Fisher Scientific, Life Technologies Corporation) was used to stain the nuclei according to the manufacturer’s instructions. As a control for immunofluorescence, the primary antibody was omitted and no fluorescence was detected under these conditions.

Pictures were acquired using a Leica TCS SP2 confocal microscope with a ×40 oil-immersion objective. Images were assembled in panels and puncta were counted using Fiji software (ImageJ Development Team, Universal Imaging Corporation, West Chester, PA, USA).

### Bioinformatics

The molecular interaction of Beclin-1 and c-FLIP_L_ was simulated by using the Schrödinger’s BioLuminate software (https://www.schrodinger.com/products/bioluminate). Standard procedure described in the manual for protein–protein docking was applied. The molecular model used for Beclin-1 is the crystal structure of Beclin-1 evolutionary conserved domain, PDB structure 4DDP [58]. The molecular model used for c-FLIP is the crystal structure deposited in PDB as 3H13 [59]. Image of the simulated complex between Beclin-1 and c-FLIP was generated with the Discovery Studio software [60] on the monomeric proteins and on the complex.

### Statistical analysis

All statistical analysis was performed using Prism software (GraphPad Software, San Diego, CA, USA). Values are expressed as mean, with individual experiments data points plotting. The statistical significance was determined by performing two-tailed Student’s *t*-test, one-way or two-way analysis of variance, depending on the number of variables involved in each experiment; a value of *P* ≤ 0.05 was considered statistically significant.

## Supplementary information

Supplementary Figure S1

Supplementary Figure S2

Supplementary Figure S3

Supplementary Figure S4

supplementary figure legends
