# Supplementary figures and images for: c-FLIP regulates autophagy by interacting with Beclin-1 and influencing its stability
Source: Cell Death Dis. 2021 Jul 8;12(7):686. doi: 10.1038/s41419-021-03957-5 (PMC8266807; doi:10.1038/s41419-021-03957-5)

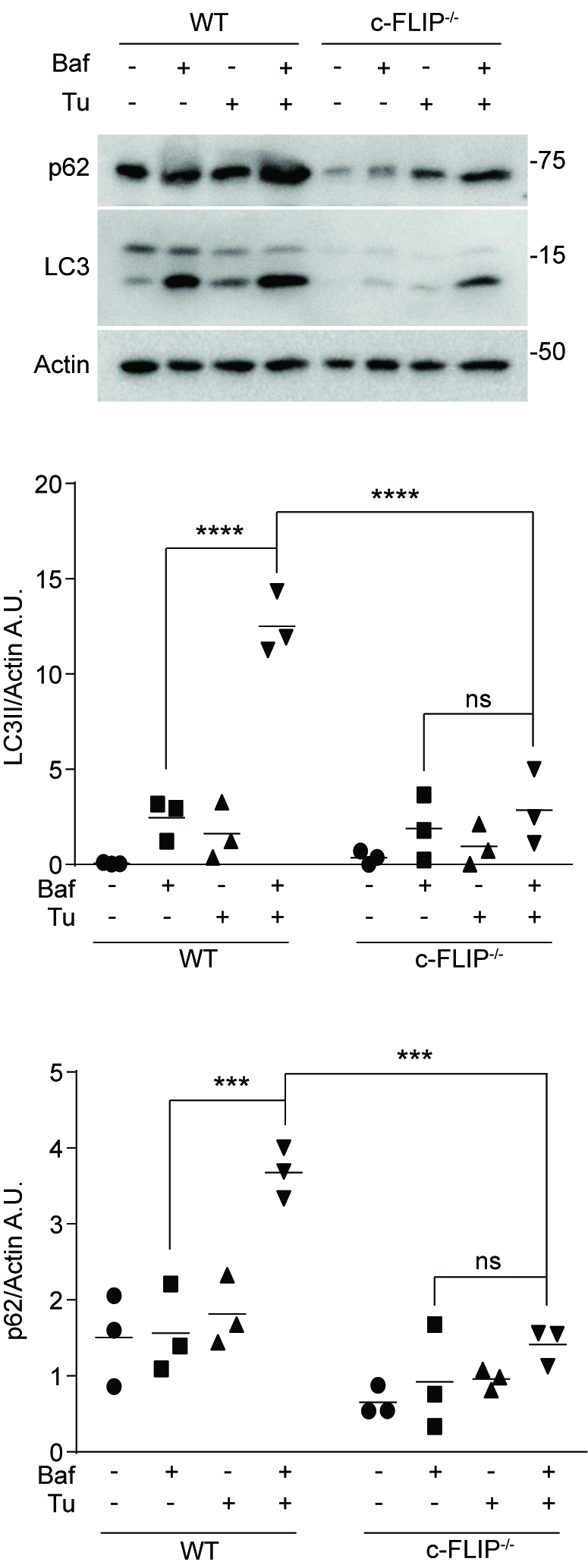

Supplement: Supplementary file 1 — Supplementary Figure S1 [file 41419_2021_3957_MOESM1_ESM.tif]

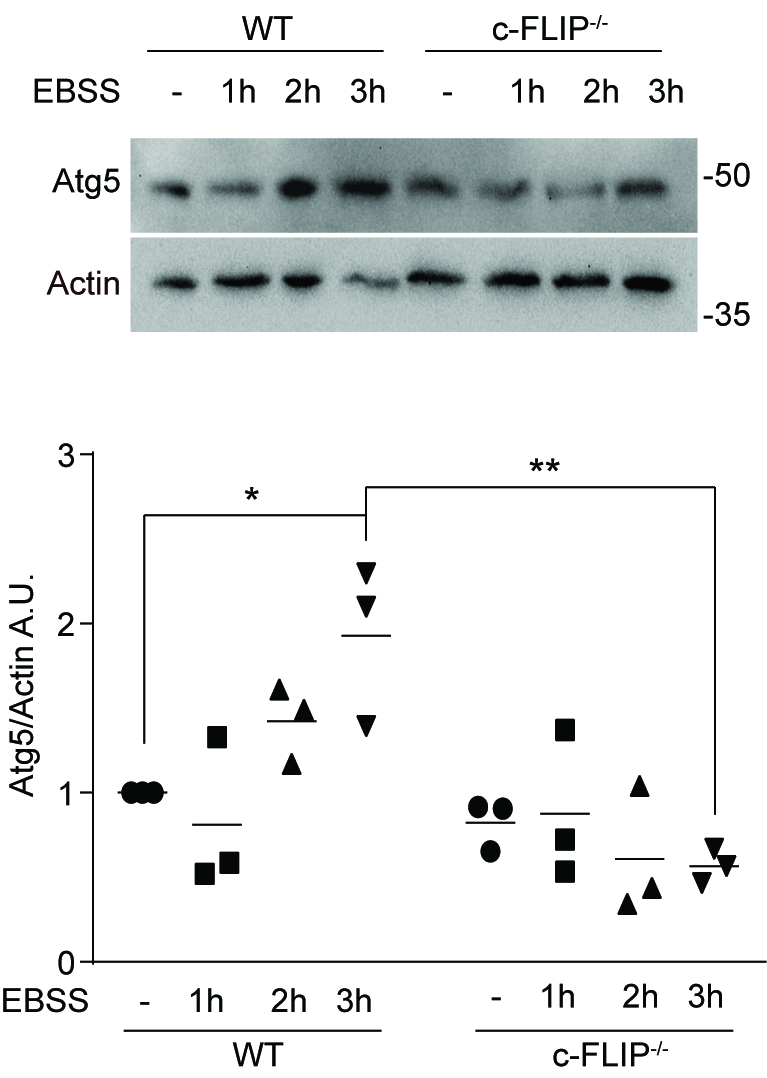

Supplement: Supplementary file 2 — Supplementary Figure S2 [file 41419_2021_3957_MOESM2_ESM.tif]

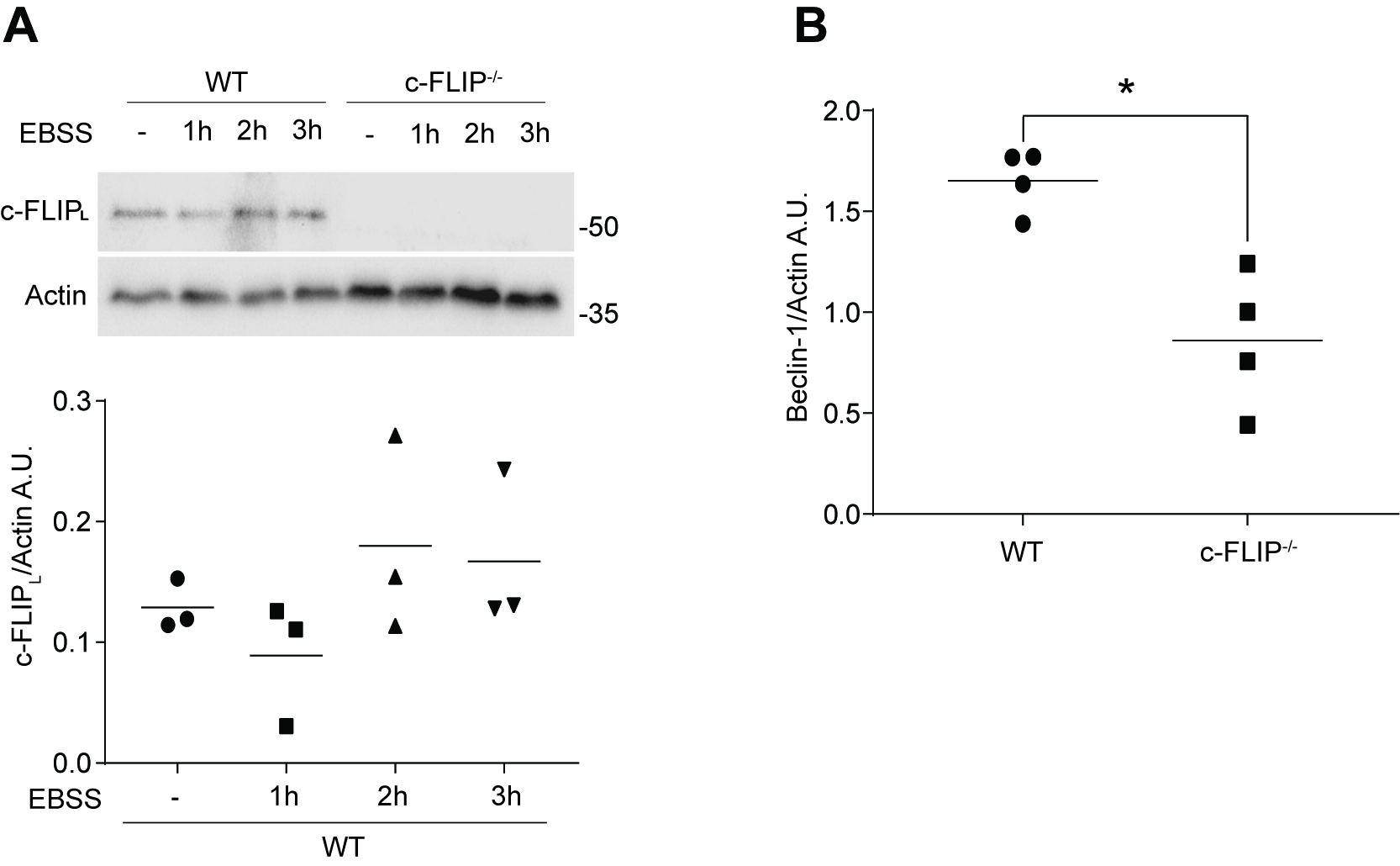

Supplement: Supplementary file 3 — Supplementary Figure S3 [file 41419_2021_3957_MOESM3_ESM.tif]

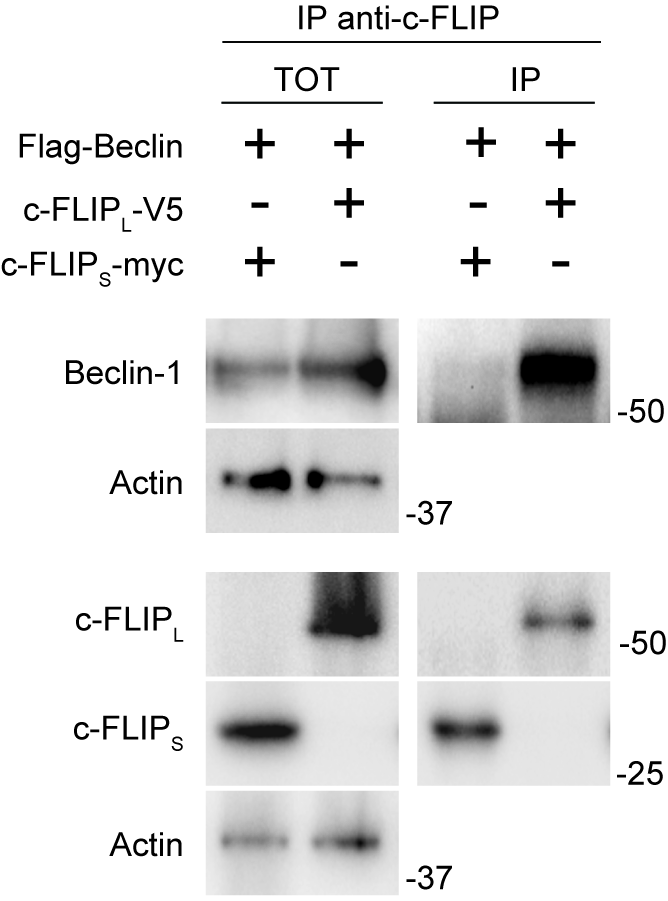

Supplement: Supplementary file 4 — Supplementary Figure S4 [file 41419_2021_3957_MOESM4_ESM.tif]
