# Supplementary material for: c-FLIP regulates autophagy by interacting with Beclin-1 and influencing its stability
Source: Cell Death Dis. 2021 Jul 8;12(7):686. doi: 10.1038/s41419-021-03957-5 (PMC8266807; doi:10.1038/s41419-021-03957-5)
Supplement: Supplementary file 5 — supplementary figure legends [file 41419_2021_3957_MOESM5_ESM.docx]

**Figure S1**

WT and *c-FLIP-/-* MEFs were treated with with tunicamycin (Tu) (1μg/ml) for 18h. Bafilomycin A1 (Baf) (100nM) was added 4h before cell lysis. Autophagic flux was assessed by measuring LC3 II and p62 protein levels by western blot. β-Actin was used as a loading control. Data shown are representative of at least three individual experiments. ****p* < 0.001; *****p* < 0.0001 determined by two-way ANOVA.

**Figure S2**

WT and *c-FLIP-/-* MEFs were cultured in EBSS for increasing times (1h, 2h, 3h) and then Atg5 expression levels were evaluated by western blot. β-Actin was used as a loading control. Data shown are representative of three individual experiments. **p* < 0.05; ***p* < 0.01 determined by two-way ANOVA.

**Figure S3**

(A) WT and *c-FLIP-/-* MEFs were cultured in EBSS over time (1h, 2h, 3h) and c-FLIP_L_ expression levels were evaluated by western blot. β-Actin was used as a loading control. Data shown are representative of three individual experiments. (B) WT and *c-FLIP-/-* MEFs were cultured and then Beclin-1 expression levels were evaluated by western blot in untreated conditions. Data shown are representative of at least three individual experiments. **p* < 0.05 determined with two-tailed Student’s *t* test.

**Figure S4**

Hek293 cells were transfected to overexpress c-FLIP_L_ (c-FLIP_L_-V5), c-FLIP_S_ (c-FLIP_S_-myc) and Beclin-1 (Flag-Beclin). An α-FLIP antibody was used to immunoprecipitate c-FLIP. The result was detected by western blot. β-Actin was used as a loading control. Data shown are representative of at least three individual experiments.
